# The pathologic response of resected synovial sarcomas to hyperthermic isolated limb perfusion with melphalan and TNF-α: a comparison with the whole group of resected soft tissue sarcomas

**DOI:** 10.1186/1477-7819-11-185

**Published:** 2013-08-12

**Authors:** Benjamin Schwindenhammer, Lars Erik Podleska, Andrea Kutritz, Sebastian Bauer, Sien-Yi Sheu, Georg Taeger, Kurt Werner Schmid, Florian Grabellus

**Affiliations:** 1 Institute of Pathology and Neuropathology, University Hospital of Essen and Sarcoma Center at the West German Cancer Center (WTZ), University of Duisburg-Essen, Hufelandstrasse 55, 45122, Essen, Germany; 2 Department of Trauma Surgery (Musculoskeletal Surgical Oncology), University Hospital of Essen and Sarcoma Center at the West German Cancer Center (WTZ),University of Duisburg-Essen, Essen, Germany; 3 Department of Internal Medicine (Cancer Research), University Hospital of Essen and Sarcoma Center at the West German Cancer Center (WTZ), University of Duisburg-Essen, Essen, Germany

**Keywords:** Isolated limb perfusion, Soft tissue sarcomas, Synovial sarcomas, Tumor regression, Tumor response

## Abstract

**Background:**

Hyperthermic isolated limb perfusion with tumor necrosis factor-α and melphalan (TM-HILP) has been successfully used to treat limb soft tissue sarcomas (STSs) with high response rates. The data on the effectiveness of HILP-TM for the treatment of STSs are mainly based on various STS types. The aim of this study was to investigate the responses of synovial sarcomas (SS) to TM-HILP.

**Methods:**

A total of 125 TM-HILP-treated tumors (STS^all^), including 14 SSs, were included in the study. The tumors were subdivided into proximal and distal limb localizations. Tumor typing (using the WHO classification), resection status (using the UICC classification), and response to therapy were assessed using light microscopy. The SSs were tested for the *SYT-SSX* translocation using RT-PCR. The following tests were applied: a chi-squared test, a *t* test, and the Mann-Whitney *U* test.

**Results:**

The SSs were localized distally more often than were the STS cohort (STS^−SS^) (85.7% vs. 32.4%) and were smaller (5.8 cm vs. 10.7 cm). There were no differences in the responder/nonresponder ratios or the mean percentages of pathological regression between the SS and STS^−SS^ cohorts (74.0% vs. 76.0%). A general localization-dependent difference in the tumor responses to TM-HILP could not be detected in the STS^all^ cohort (distal, 72.0% vs. proximal, 78.0%); however, a UICC R0 status was more often observed in proximal tumors (distal, 50.0% vs. proximal, 71.4%). There was no association between the *SYT-SSX* type and SS responses to TM-HILP.

**Conclusions:**

Because of the high response rates, TM-HILP is recommended for the treatment of SSs. The distal limb localization of TM-HILP-treated STSs was generally (STS^all^ cohort) associated with fewer R0 resections.

## Background

Soft tissue sarcomas (STSs) are members of a heterogeneous group of tumors that encompasses more than 50 subtypes. The majority of STSs are located in the limbs. The mainstay of local treatment for sarcomas of the extremities is limb-sparing surgery with complete tumor resection and clear surgical margins [1].

Hyperthermic isolated limb perfusion with tumor necrosis factor-α (TNF-α) and melphalan (TM-HILP) is a promising local treatment for locally advanced STSs, such as nonresectable STSs of the limbs, and has resulted in high tumor response rates and high limb salvage rates [2].

STSs represent a heterogeneous group of malignancies with different tumor biology and prognoses; however, the pathological grading, clinical evaluation and treatment of STS types follow common principles. Published data on the effectiveness and outcomes of TM-HILP-treated STSs are based on mixed STS cohorts: undifferentiated sarcomas and liposarcoma subtypes represent the majority of cases. In these studies, only sporadic histopathological regression data were obtained after TM-HILP, and the SS regression results are presented for only small numbers of cases and with variable results (Table 1).

**Table 1:** Regression of SSs in other isolated limb perfusion studies with complete histopathological regression data

**Study**	***N***	**Result (regression %)**	**Valuation**
[3]	24	Not specified for SS	-
[4]	24	Not specified for SS	-
[5]	14	Not specified for SS	-
[6]	10	Not specified for SS	-
[7]	9	Not specified for SS	-
[8]	6	Not specified for SS	-
[9]	6	Mean 66%; median 75%	Modest
[10]	5	2 × >90%; 3 × 90% to 60%	Modest
[11]	5	2 × 80%, 1 × 10, 35, 70%	Modest
[12]	4	3 × >90%; 1 × 10 to 50%	Good
[13] and [14]	4	3 × ≥50%; 1 × < 50%	Modest
[15]	4	3 × 0%; 1 × 25%	Poor
[16]	4	3 × ≤50%; 1 × >50%	Poor
[17]	2	1 × 100%, 1 × >80%	Good
[18]	2	1 × 80%; 1 × 95%	Good
[19]	2	Not specified for SS	-
[20]^a^	1	1 × >50%	-

Studies with complete histopathological regression data or a report of SS regression were included; ^a^TM-HILP + interferon-γ.

The aim of this study was to investigate the STS subgroup of synovial sarcomas (SSs) and their histopathological responses to TM-HILP and to compare the results with those for the complete TM-HILP-treated STS cohort (STS^−SS^) and with those found in the literature.

## Methods

### Patients

From February 2002 to November 2012, 164 patients with STSs (including 20 with SS) were treated with TM-HILP at the University Hospital of Essen.

Resection specimens could be obtained from 125 (76.2%) patients (STS^all^), including 14 patients with SSs, for whom TM-HILP was performed to treat a nonresectable soft tissue sarcoma manifestation.

Patients were selected for the TM-HILP program according to the inclusion criteria outlined by Eggermont *et al*. [21]. Patient evaluation and TM-HILP treatment were exclusively performed by the same specialized team. In brief, TM-HILP was performed under mild hyperthermia (39°C) and leakage monitoring with radiolabeled serum. The administered dose of recombinant human TNF-α was adjusted to 0.25 mg/l perfused tissue volume (but not less than 1 mg/limb), and melphalan (L-phenylalanine mustard) was used at concentrations of 11 mg/l for legs and 13 mg/l for arms.

All the patients provided informed consent, and the study was performed strictly according to the Declaration of Helsinki.

### Gross pathology

All the specimens were fixated in 4% neutral buffered formalin. For all the specimens, a gross estimation of tumor necrosis was recorded and a minimum of one block per centimeter of the largest dimension of the tumor was collected.

### Histopathology

Basic microscopic evaluation of H&E-stained slides was performed.

#### Typing and grading

The STSs were typed according to the World Health Organization (WHO) classification of tumors for STSs and bone tumors. The STSs were graded using primary biopsies according to the French Fédération nationale des centres de lutte contre le cancer. During subtyping, the SSs were further divided into spindle cell (monophasic fibrous), biphasic, and poorly differentiated types [22].

#### Tumor regression after therapy

**Figure 1:**
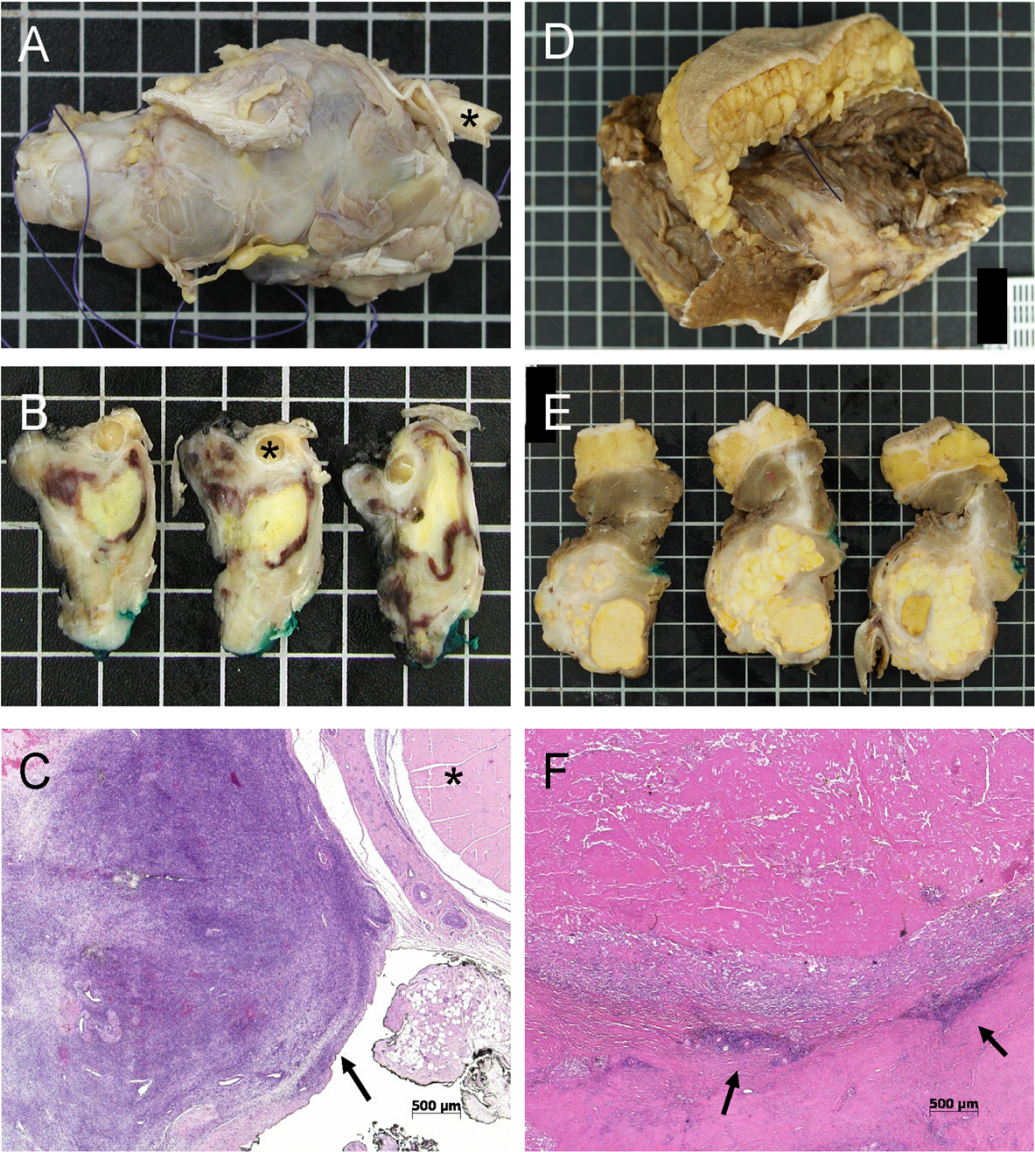
**Macroscopic and microscopic features of two SSs after treatment with TM-HILP. (A-C)** Case number 5, nonresponder. **(A)** 7.9 cm SS of the foot with a regression of 65% after TM-HILP. **(B)** Necrotic areas with yellow tumor changes and viable tumor residues in proximity to a removed tendon segment (*). **(C)** Histopathology of a predominately viable tumor next to the cross-section of a tendon (*). The tumor infiltrates the inked margin (arrow). **(D-F)** Case Number 6, responder. **(D)** 9.0 cm SS of the lower leg with a regression of 99%. **(E)** Abundant areas of yellow necrosis mixed with glassy-white sclerotic areas after therapy. **(F)** Histopathology revealed only a few small foci of viable tumor (arrows). These residues were completely removed.

Total regression was assessed using light microscopy (Figure 1) and was determined as the percentage of the devitalized tumor after TM-HILP. Tumor regression was graded using the six-stage grading scale of Salzer-Kuntschik [23], and the tumors were further subdivided into ‘responders’ (<10% viable tumor) and ‘nonresponders’ (≥10% viable tumor).

#### Reverse transcriptase polymerase chain reaction (RT-PCR)

For RT-PCR, three or four 10-μm thick sections of tumor tissue were transferred to microcentrifuge tubes and deparaffinized. RNA extraction was conducted using the RNeasy FFPE kit (QIAGEN, Hilden, Germany). For complementary deoxyribonucleic acid (cDNA) synthesis, RT-PCR was performed using M-MulV reverse transcriptase (Fermentas, St Leon-Rot, Germany). The presence of amplifiable mRNA was confirmed by RT-PCR using control primer sets for hypoxanthine-phosphoribosyl-transferase. The cDNA products were then subjected to gene fusion experiments by PCR (Primers SYT-F: AGACCAACACAGCCTGGACCA; SSX1-R: GGTGCAGTTGTTTCCCATCG; and SSX2-R: GGCACAGCTCTTTCCCATCA [24]. Finally, the PCR products were visualized using electrophoresis with 2% agarose gels and ethidium bromide staining. The DNA from *SYT-SSX1-* and *SYT-SSX2-*positive SSs served as positive controls.

### Statistics

Statistical analyses were performed with IBM SPSS Statistics 19. The following tests were applied: a chi-squared test, a *t* test, and the Mann-Whitney *U* test. *P* values ≤ 0.05 were considered statistically significant.

## Results

### Patients

The male-to-female ratios were 6:8 for the synovial sarcoma (SS, Table 2) cohort and 52:59 for the complete STS cohort (STS^−SS^). The mean age of the SS patients was lower than that of the STS^−SS^ patients (45 vs. 57 years; *t* test, *P* < 0.01). The SSs were mainly primary tumors (8/14, 57.1%). Five tumors were recurrences, and one tumor was a residuum after prior surgery (STS^−SS^: 86 primary tumors, 77.5%; 22 recurrences, 19.8%; and 3 residua, 2.7%). Moreover, the tumors in the SS cohort were more often localized distally (proximal/distal: SS, 2/12, 85.7% vs. STS^−SS^, 75/36, 32.4%; chi-squared test, *P* < 0.001). Distal localization was defined distally relative to the knee or elbow joint. The foot was the primary localization of the SS cohort (5/14, 35.7%), and the thigh was the most frequent site of the STS^−SS^ cohort (60/111, 54.1%).

**Table 2:** Characteristics of the synovial sarcomas

**Tumor number**	**Age of patient**	**Sex of patient**	**SS type**	***SYT-SSX *fusion type**	**Localization**	**Size (cm)**	**Regression (%)**
1	52	Female	Spindle cell	*SSX2*	Foot	3.5	99
2	44	Male	Spindle cell	*SSX1*	Forearm	3.2	3
3	24	Female	Spindle cell	*SSX2*	Lower leg	2.5	85
4	24	Male	Spindle cell	*SSX1*	Thigh	6.2	98
5	75	Female	Biphasic	*SSX1*	Foot	7.9	65
6	29	Female	Biphasic	*SSX2*	Lower leg	9.0	99
7	38	Female	Spindle cell	*SSX1*	Thigh	16.0	20
8	65	Male	Spindle cell	*SSX1*	Foot	5.5	80
9	70	Female	Biphasic	*SSX1*	Forearm	3.5	100
10	46	Male	Spindle cell	*SSX1*	Lower leg	7.0	30
11	10	Female	Poorly differentiated	*Wild type*	Foot	4.0	100
12	45	Male	Biphasic	*SSX1*	Lower leg	10.0	65
13	58	Male	Spindle cell	*SSX1*	Foot	3.0	99
14	50	Female	Spindle cell	*SSX1*	Forearm	1.2	98

### Pathology

#### Basic parameters

The most frequent STS subtypes of the STS^−SS^ cohort were the undifferentiated pleomorphic sarcoma (28/111, 25.2%) and liposarcoma types (23/111, 20.7%). The tumor grades according to the French Fédération nationale des centres de lutte contre le cancer (three grades: 1, 2, and 3) were 0, 8, and 6 for the SS cohort and 7, 49, and 55 for the STS^−SS^ cohort. Moreover, the SSs were significantly smaller at the time of resection after TM-HILP (mean size: SS, 5.8 cm vs. STS^−SS^, 10.7 cm; Mann-Whitney *U* test, *P* = 0.004).

#### Regression after TM-HILP

**Figure 2:**
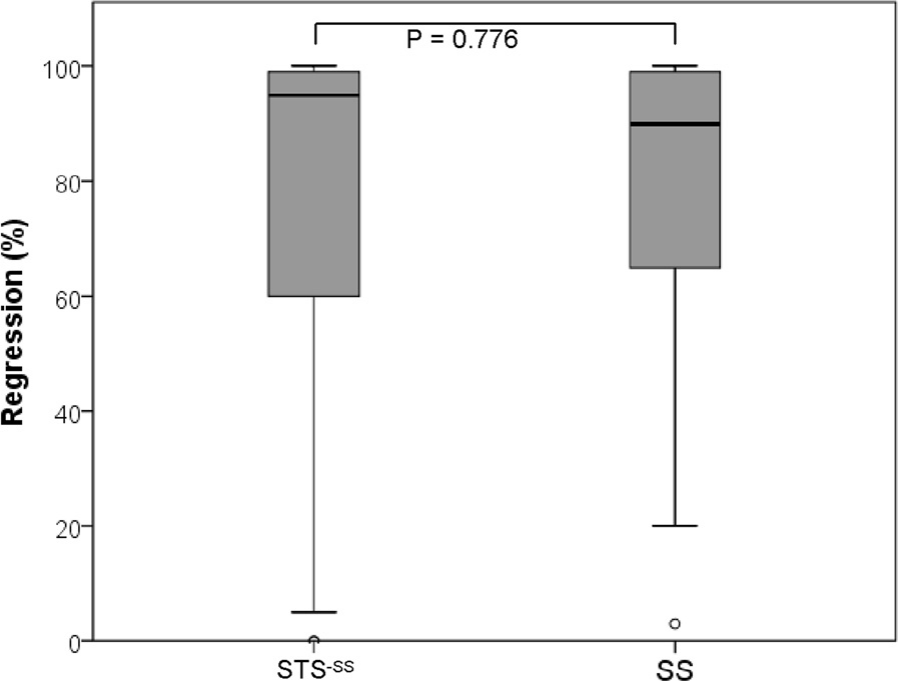
**Box plots show a comparison of the regression results (%) between the synovial sarcomas (SS, *N *= 14) and the comparison group (STS**^**−SS**^**, *N *= 111).** No significant differences were detected.

There were 7/14 (50.0%) responders in the SS cohort according to the Salzer-Kuntschik regression grading scale. Two (14.3%) of those SSs exhibited complete pathological regression. The STS^−SS^ cohort included 66/111 (59.5%) responders, of which 24 (21.6%) were complete responders. There were no differences in the responder/nonresponder ratios (chi-squared test, *P* = 0.499) or in the mean percentages of pathological regression between the SS and STS^−SS^ cohorts (SS, 74.0% vs. STS^−SS^, 76.0%; Mann-Whitney *U* test, *P* = 0.776, Figure 2).

Because of the predominant distal localization of the SSs in this study, we did not detect any localization-dependent (distal/proximal) differences in the tumor responses to TM-HILP according to the mean percentages of pathological regression in the STS^all^ cohort (distal, *N* = 48, 72.0% vs. proximal, *N* = 77; 78.0%; Mann-Whitney *U* test, *P* = 0.493).

#### The *SYT-SSX* fusion type and responses

Overall, ten SS cases were *SYT-SSX1-*positive and three were *SYT-SSX2*-positive. In one case (see Table 1), none of the fusions could be detected by PCR. Despite the negative molecular pathological results for *SYT-SSX1* and *SYT-SSX2,* this tumor was typed as SS by three sarcoma experts at three different institutions because of its conventional, immunohistochemical characteristics and a borderline fluorescence *in situ* hybridization result.

The SSs with these genetic signatures included both responders and nonresponders (see Table 1), and there were no significant differences among the responses to TM-HILP. The *SYT-SSX2* fusion type was exclusively found in female patients (3/3).

### Resectability

The investigation of the resection status (UICC, R classification) found 8/14 (57.1%) complete resections (R0 status) in the SS cohort. Both complete responders (100% pathological devitalization) in the SS group had an R1 status. Complete resection was observed in 71/111 (64.0%) STS^−SS^ tumors. This difference between the groups was not statistically significant (chi-squared test, *P* = 0.618). Both proximal SSs (2/2) and 6/12 of the distal SSs were completely resected, which is principally in line with the results below, but was not significant for the SS group alone. However, the resection status results of the proximal and distal limb localizations indicated better resectability for proximal STSs in the STS^−SS^ cohort according to the R0 status (53/71 R0 proximal, 74.6% vs. 22/40 R0 distal, 55.0%; chi-squared test, *P* = 0.034). This result remained statistically significant when the 14 SSs were included in the calculation (STS^all^: 55/77, 71.4% vs. 24/48, 50.0%; chi-squared test, *P* = 0.016).

## Discussion

The treatment of STSs is increasingly determined by the identification of histological subtypes in a family of uncommon cancers that comprises more than 50 subtypes [25]. Published data on the effectiveness and outcomes of TM-HILP-treated STSs are based on studies in which common and rare STS entities were mixed [2]. Histology is relevant for target therapies and now for the precise application of conventional chemotherapy regimens; therefore, we suggest that the effect of TM-HILP on STS subentities should be confirmed.

### The frequencies and sizes of the SSs

SSs represent approximately 5% to 10% of all STSs. Because of their predominant localization within the limbs, the frequency of SSs at limb sites has ranged from 12% to 15% of all limb STSs [26]. The frequency of SSs in this investigation was 11.2% in a limb STS cohort, which is within the range of previously published data. Additionally, we observed a smaller median tumor size (4.2 cm) than that reported for large SS cohorts (6 to 7 cm) [27-30]. This finding may be a consequence of the various tumors in this study, which included primary tumors (5.5 cm) and recurrences and residual tumors after surgery (3.7 cm).

### Localization of the SSs

Most SSs are limb-based tumors. Approximately 67% to 86% of SSs arise within the extremities [27-31]. Of those SSs, distal localization was found in 47% to 71% [27,29,31] of cases, which is approximately twice as high as the rates for limb STSs [32,33]. Additionally, a higher rate was found in this study (86.7%), which is a hallmark of isolated limb perfusion (ILP) studies.

### Responses of SSs to TM-HILP

The clinical and pathological response rates of sarcomas to TM-HILP are typically high. The mean clinical overall response in TM-HILP studies according to the WHO criteria [34] was initially reported to be more than 90% with complete responses in more than 80% of cases [35]. To date, the largest studies have adjusted these clinical data downward to an overall response rate of approximately 70% and a complete response rate of 20% [36]. Interestingly, clinical and pathological response evaluations have often differed substantially, which we have recently demonstrated to be the result of an insufficiency in size-based clinical response criteria that would indicate actual tumor responses (measured by a pathological investigation as the gold standard) [37]. The SS cohort exhibited a mean pathological response rate that was approximately identical to that of the STS^−SS^ in this cohort of resected STS. This indicates that TM-HILP can be recommended for this STS subtype despite the disproportionate number of distal limb localizations.

### Responses of the genetic subtypes to TM-HILP

The t(X;18) translocation, which results from the *SYT-SSX1* and *SYT-SSX2* gene fusions*,* is found in approximately all SSs [26]. This genetic alteration has not yet been found in any other human malignancy; therefore, these genetic signatures have significant diagnostic value. The impact of the *SYT-SSX* fusion type on the clinical behavior of SSs is the subject of scholarly discourse with contrasting results [28,38]. The influence of the translocation type on the response to treatment has not been investigated to date but might be reflected by the prognostic significance of the type according to metastatic potential and overall survival times [38,39]. This study did not find varying responses to TM-HILP among the *SYT-SSX* translocation types in our small SS cohort.

### Resectability of the SSs after TM-HILP

The mainstay of surgical treatment for STSs is limb-sparing resection with clear surgical margins [1]. There is disagreement regarding the size of an adequate wide surgical margin [1]. TM-HILP is indicated only for the subgroup of STS patients who are candidates for disabling surgery or amputation. In this study, a surgical approach with oncological adequacy of margin width was not possible. Even after TM-HILP, the margin size and quality is generally poor [40]. However, we have previously demonstrated that local tumor control after TM-HILP and resection is superior compared with nonpretreated STSs, most probably due to the strong toxic effects of TM-HILP on the tumor periphery [3,40].

This study demonstrated a high rate of distally located tumors (40%), which is typical for ILP-treated STS cohorts compared with most non-ILP-treated cohorts. Because of the specific anatomic conditions that are found in the distal parts of the extremities, the rate of complete resections with clear surgical margins is generally low [41]. Tumors are frequently rated as ‘primarily unresectable’ and are possible candidates for TM-HILP programs. Several large studies have demonstrated proximal and distal localizations of STSs but only provided the histological margin status for the complete cohort (≈10% to 20% R1-resections) [33,42,43]. Studies that have selectively investigated ‘distal STSs’ have not included histopathological data concerning the surgical margins [44-48].

This study found a comparatively high rate of incompletely resected tumors after TM-HILP (38.4%), which is in agreement with results from studies in Rotterdam [15,36]. In addition, this study found a significantly reduced resectability (in terms of the R0 classification) rate in distal limb localizations [15]. Interestingly, the average width of the margins of the R0 resected tumors did not vary significantly according to localization and was low: the proximal mean was 0.8 mm, and the distal mean was 1.2 mm.

A worsening of the effectiveness of TM-HILP on distally located STSs, possibly due to impaired blood flow, was not detected in our study. Deroose *et al*. [15] recently investigated STSs of distal limbs and found a limb salvage rate of 87% after TM-HILP. They stated, ‘TM-ILP is an effective treatment modality in patients with distal STS,’ but they found a considerably lower average percentage of pathological regression than that in this study (33.4% vs. 70.0%).

## Conclusion

In conclusion, we found that the preoperative treatment TM-HILP is as effective for the subentity of SSs as for the complete group of STSs. Accordingly, TM-HILP is recommended for this tumor type even though it is often located distally. A distal localization resulted in reduced resectability in the STS^all^ cohort according to the R0 rate, but the margin size of the complete resections did not differ significantly between both types of localization.

## Abbreviations

H & E: hematoxylin and eosin; ILP: isolated limb perfusion; RT-PCR: reverse transcriptase polymerase chain reaction; SS: synovial sarcomas or cohort of SS of this study; STS: soft tissue sarcomas; STSall: all soft tissue sarcomas of the present study (SS included); STS−SS: soft tissue sarcomas of the present study, excluding SS (comparison group); TM-HILP: hyperthermic isolated limb perfusion with TNF-α and melphalan; TNF-α: tumor necrosis factor-α; UICC: Union for International Cancer Control; WHO: World Health Organization.

## Competing interests

The authors declare that they have no competing interests.

## Authors’ contributions

BS, LEP, AK, GT and FG made substantial contributions to conception and design, or acquisition of data, or analysis and interpretation of data. BS was responsible for histopathology. LEP and GT were responsible for the clinical data. AK was responsible for genetic testing. FG was responsible for histopathology and statistics. BS, LEP, SS and FG were involved in drafting the manuscript or revising it for important intellectual content. BS and KWS designed the study and drafted the manuscript. All authors read and approved the final manuscript.
